# Regularized gene selection in cancer microarray meta-analysis

**DOI:** 10.1186/1471-2105-10-1

**Published:** 2009-01-01

**Authors:** Shuangge Ma, Jian Huang

**Affiliations:** 1 Department of Epidemiology and Public Health, Yale University, New Haven, CT 06520, USA; 2 Department of Statistics and Actuarial Science, University of Iowa, Iowa City, IA 52242, USA

## Abstract

**Background:**

In cancer studies, it is common that multiple microarray experiments are conducted to measure the same clinical outcome and expressions of the same set of genes. An important goal of such experiments is to identify a subset of genes that can potentially serve as predictive markers for cancer development and progression. Analyses of individual experiments may lead to unreliable gene selection results because of the small sample sizes. Meta analysis can be used to pool multiple experiments, increase statistical power, and achieve more reliable gene selection. The meta analysis of cancer microarray data is challenging because of the high dimensionality of gene expressions and the differences in experimental settings amongst different experiments.

**Results:**

We propose a Meta Threshold Gradient Descent Regularization (MTGDR) approach for gene selection in the meta analysis of cancer microarray data. The MTGDR has many advantages over existing approaches. It allows different experiments to have different experimental settings. It can account for the joint effects of multiple genes on cancer, and it can select the same set of cancer-associated genes across multiple experiments. Simulation studies and analyses of multiple pancreatic and liver cancer experiments demonstrate the superior performance of the MTGDR.

**Conclusion:**

The MTGDR provides an effective way of analyzing multiple cancer microarray studies and selecting reliable cancer-associated genes.

## Background

Microarrays are capable of profiling human tissues on a genome-wide scale and have been used extensively in cancer studies, where expressions of thousands of genes are measured along with clinical outcomes. A major goal of such studies is to identify a subset of cancer-associated genes that can be used as biomarkers for cancer diagnosis and prognosis and as targets for therapy. Early studies have shown that gene signatures identified from the analysis of individual cancer microarray experiments often have low reproducibility. There are several reasons for this. A main one is that the sample size of a single microarray experiment, which is usually in the hundreds, is much smaller than the number of genes, which is usually in the tens of thousands.

Within the field of clinical investigation, meta analysis has emerged as the gold standard for the comparison and combined analysis of clinical studies. It is generally accepted that only meta analysis can circumvent the problems inherent to studies with low statistical powers due to low sample sizes [1]. With meta analysis, it is usually not the intention of researchers to analyze any new datasets. Rather, it provides an effective way of pooling and analyzing multiple existing datasets and generating results more reliable than those from the analysis of each individual data set.

Meta analysis of cancer microarray data is made possible by the many experiments conducted independently to measure the same set of genes and the same cancer clinical outcomes. As shown in [2-5], the meta analysis of cancer microarray data has achieved considerable successes by identifying relatively reproducible, biologically meaningful gene signatures. We refer to [6] for more discussions of the merits of meta analysis in genomic studies.

Meta analysis of cancer microarray data is challenging because (1) microarray experiments usually measure a small number of samples and a large number of genes, with only a subset of those genes associated with cancer clinical outcomes. Gene selection is needed along with estimation; (2) the meta analysis of cancer microarray data and the identification of cancer-associated genes often require the use of original expression measurements. For this reason, the type of analysis conducted in this article has also been referred to as "integrative analysis". Such analysis differs significantly from conventional meta analysis, where the analysis is based on summary statistics (such as p-values) from each individual experiment; and (3) different platforms may be used in different experiments. Arrays that hybridize one sample at a time (e.g., synthesized oligonucleotide arrays) measure gene expression based directly on the signal intensity of each probe set. In contrast, spotted cDNA arrays hybridized with fluorescent-labeled targets typically measure the ratio of the signal from a test sample to the signal of a co-hybridized reference sample. It has been shown that data from Affymetrix GeneChip oligonucleotide microarrays correlate poorly with the data from custom-printed cDNA microarrays [7]. We note here that comparability of different platforms can be achieved by the transformation of the expressions. However, as noted in previous studies (such as [8]), such transformation needs to be conducted on a case-by-case basis.

Several approaches have been proposed to analyze the marginal effects of genes using data from multiple microarray experiments. Examples of this include Fisher's approach (with application to breast cancer [9]); an intensity approach that transforms and directly integrates gene expressions [5]; a penalization approach [3]; a random effect model based approach [10]; a robust gene ranking approach [11]; and a Bayesian approach [12].

In light of the fact that cancer development and progression are caused by the effects of multiple genes, the following studies (which can account for the joint effects of genes) have been conducted. A majority voting (with impact factors) approach has been proposed by [13]. Gene shaving approaches based on random forrest and Fisher's linear discrimination are applied in [14]. And a computationally intensive Bayesian approach is proposed in [15]. We note that the focus of those studies has been predictive model building, not gene selection.

On the other hand, there is rich literature for the analysis of a single cancer microarray data and gene selection. Examples include the parameterized classifier design approach in [16]; the penalization approaches in [17,18]; the Threshold Gradient Directed Regularization (TGDR) approach [19-21]; and the support vector machine approach [22]. We refer to [23] for more discussions of gene selection approaches with individual microarray datasets. We note, however, that those approaches have been designed to analyze a single dataset, and cannot be used to analyze multiple, heterogeneous datasets.

The literature review suggests that (1) genes identified from analysis of a single cancer microarray data may suffer from low reproducibility because of the small sample size. Meta analysis pools multiple datasets, increases statistical power, and provides an effective way of improving reproducibility; (2) existing meta analysis approaches focus on either the investigation of the marginal effects of genes or the construction of predictive models with multiple genes; and (3) approaches exist that can select genes with joint effects on cancer in the analysis of a single dataset. However, these approaches cannot be used to analyze multiple, heterogeneous data. Thus, there is a critical need for approaches that can select genes with joint effects on cancer in the meta analysis of multiple microarray data.

In this article, we propose the Meta Threshold Gradient Descent Regularization (MTGDR) approach for gene selection in cancer microarray meta analysis. The MTGDR takes advantage of recent developments in regularized gene selection with a single microarray dataset. Compared to such single-dataset gene selection methods, the MTGDR has the desired flexibility of accommodating multiple experiments with different setups. And in comparison with the available meta analysis methods, the MTGDR can effectively select a subset of genes with joint effects on cancer.

## Results and discussion

### Simulation study

We conduct simulation studies to investigate the performance of the proposed MTGDR. We generate *M *= 3 datasets. For dataset *m *= 1, 2 and 3, we generate *n*_*m *_samples and expressions of *d *genes. Gene expressions are generated in a way that all expressions have marginally normal distributions with unit variance, and the correlation between the expressions of genes *i *and *j *is 0.4^|*i*-*j*|^. In each dataset, the first 20 genes are associated with the cancer outcome. Specifically, for genes *i *= 1, ..., 20, the mean expressions of the *n*_*m*_/2 cases (outcome *Y*_*m *_= 1) are generated randomly from *Uniform*[*l*, *u*]. The mean expressions for the genes of the controls (outcome *Y*_*m *_= 0) are zero. The mean expressions for the genes not associated with the outcomes are zero. The simulation setting here corresponds to the logistic regression models for all three datasets. The regression coefficients for the cancer-associated genes vary across studies, which corresponds to different experimental setups (for example different platforms) in different studies.

We consider combinations of the following simulation settings: (1) sample size *n*_*m *_= 30 and 100; (2) number of genes *d *= 100, 500 and 1000; and (3) different levels of "signals" [*l*, *u*] = [0.5, 1.0] and [1.0, 1.5]. Thus, there are a total of 12 different simulation scenarios.

We employ the proposed MTGDR, and tuning parameters are selected via the 3-fold cross validation. For comparison, we also consider the following two alternative approaches: (1) the pooled TGDR approach. Other than the differences in regression coefficients (shifts of mean expressions), the three datasets are generated in a comparable manner. We pool all three datasets together, treat them as if they were from a single experiment, and analyze them with the TGDR approach; and (2) the meta analysis approach based on individual TGDR analysis. We first analyze each dataset using the TGDR approach. We then search for genes identified in all three studies. This corresponds to the meta analysis approach where each dataset is analyzed separately using the TGDR and the results are combined via a voting approach. We note that other alternative approaches exist. For example, it is possible to replace the TGDR approach with the penalization approaches discussed in [23]. Early studies have established the comparable performance of the TGDR with alternative approaches [19-21]. Since the proposed MTGDR shares a similar thresholding paradigm with the TGDR, we focus on the aforementioned two alternatives.

In Table 1, we show the mean (standard deviation) of the number of identified genes and the number of true positives based on 200 replicates. We can see that (1) the proposed MTGDR is capable of identifying the majority of the genes truly associated with the outcome and has very small false positive rates; (2) the performance of the pooled analysis is less satisfactory but still acceptable. We note that the three simulated datasets are more comparable than those encountered in practical studies. The regression coefficients differ across datasets, though the differences are small. This comparability explains the reasonable performance of the pooled analysis and should not be expected in general with practical data; and (3) the "individual TGDR + voting" meta analysis approach has inferior performance, which is caused mainly by the small sample size and the subsequent lack of reproducibility of each individual dataset. We have also conducted simulations under other settings and drawn similar conclusions (results not shown).

**Table 1:** Simulation studies.

			Pooled TGDR		meta analysis		MTGDR	
*n*_*m*_	*d*	[*l*, *u*]	Positive	True pos.	Positive	True pos.	Positive	True pos.
30	100	[0.5, 1]	15 (2.2)	13 (1.7)	2 (1.3)	2 (1.3)	16 (1.8)	15 (1.8)
	500	[0.5, 1]	17 (2.6)	13 (1.9)	2 (1.2)	2 (1.2)	15 (1.7)	14 (1.7)
	1000	[0.5, 1]	19 (3.3)	13 (2.0)	2 (1.2)	2 (1.2)	16 (1.9)	14 (1.7)
30	100	[1, 1.5]	13 (2.0)	13 (1.9)	1 (1.0)	1 (1.0)	13 (2.0)	13 (2.0)
	500	[1, 1.5]	14 (2.1)	10 (1.8)	1 (1.0)	1 (1.0)	14 (1.7)	14 (1.7)
	1000	[1, 1.5]	14 (2.2)	12 (1.9)	1 (0.8)	1 (0.8)	15 (1.9)	14 (1.9)
100	100	[0.5, 1]	18 (1.7)	15 (1.5)	6 (1.9)	6 (1.9)	18 (1.8)	17 (1.5)
	500	[0.5, 1]	21 (2.9)	16 (1.5)	5 (1.8)	5 (1.8)	19 (2.1)	18 (1.4)
	1000	[0.5, 1]	22 (2.8)	16 (1.5)	5 (1.8)	5 (1.8)	19 (2.3)	17 (1.5)
100	100	[1, 1.5]	16 (1.5)	14 (1.5)	4 (1.5)	4 (1.5)	16 (1.7)	16 (1.7)
	500	[1, 1.5]	18 (2.2)	14 (1.7)	4 (1.6)	4 (1.6)	16 (1.7)	15 (1.6)
	1000	[1, 1.5]	14 (2.2)	12 (1.7)	4 (1.6)	4 (1.6)	17 (1.7)	16 (1.6)

Mean (standard deviation) of positive (number of genes selected by each approach) and true positive (number of selected genes that are truly cancer-associated) based on 200 replicates.

### Pancreatic cancer study

#### Data

Pancreatic ductal adenocarcinoma (PDAC) is a major cause of malignancy-related deaths. Apart from surgery, there is still no effective therapy, and even resected patients die usually within one year postoperatively. Several experiments have been conducted using microarrays to identify pancreatic cancer genomic markers. In our study, we gather and analyze four studies, which are first reported in [24-27]. These four datasets have also been analyzed by [28], and it has been argued that the clinical settings in the four studies are comparable. Thus, it is reasonable to conduct meta analysis with such data. We show the data descriptions in Table 2. Two of the four studies use cDNA arrays, and two use oligonucleotide arrays. Cluster ID and gene names are assigned to all of the cDNA clones and Affymetrix probes based on UniGene Build 161. The two sample groups considered in our analysis are PDAC and normal pancreatic tissues. Data on chronic pancreatitis are available for [25,27], but will not be used in our analysis.

**Table 2:** Pancreatic cancer study: Data information.

Dataset	P1	P2	P3	P4
Reference	Logsdon	Friess	Iacobuzio-Donahue	Crnogorac-Jurcevic
PDAC	10	8	9	8
Normal	5	3	8	5
Array	Affy. HuGeneFL	Affy. HuGeneFL	cDNA Stanford	cDNA Sanger
UG	5521	5521	29621	5794

Reference: first author of the corresponding reference; PDAC: number of PDAC samples; Normal: number of normal samples; Array: type of array used; UG: number of unique UniGene clusters.

For each dataset, data processing (including normalization) has been separately conducted by researchers in each individual study. We identity a consensus set of 2984 UniGene IDs. We remove genes with more than 30% missingness in any of the four datasets. There are 1204 genes remained for downstream analysis. For each data separately, if Affymetrix is used, we first add a floor of 10 and make log2 transformations of the expressions. We then fill in missing values with medians across samples and standardize each gene expression to have zero mean and unit variance.

#### MTGDR analysis

In the MTGDR analysis, tuning parameters are chosen via the 3-fold cross validation. Fifteen genes are identified as being associated with the risk of developing pancreatic cancer. We show the gene IDs and corresponding estimates in Table 3. We can see that if a gene has a nonzero coefficient in one dataset, then it has nonzero coefficients in all datasets (which indicates that this gene is identified in all studies). We also note that the estimated coefficients for one gene can be different across studies. This is the extra flexibility allowed by the MTGDR over the pooled analysis, which naturally accommodates differences among experimental setups in different studies. Furthermore although the estimated coefficients may be different for one gene across experiments, their signs are the same. The same signs lead to similar biological conclusions (i.e., whether up-regulations of the genes are positively or negatively associated with the risk of developing cancer).

**Table 3:** Pancreatic cancer study: MTGDR estimates and rank (in meta analysis of marginal effects).

UniGene	Gene name	P1	P2	P3	P4	Rank
Hs.107	Fibrinogen-like 1	-0.078	-0.074	-0.096	-0.062	1
Hs.12068	Carnitine acetyltransferase	-0.265	-0.387	-0.189	-0.250	7
Hs.16269	B-cell CLL/lymphoma 7B	0.038	0.055	0.060	0.017	273
Hs.169900	PABPC4	-0.879	-0.992	-0.693	-0.775	15
Hs.180920	RPS9 ribosomal protein S9	-0.144	-0.244	-0.223	-0.189	53
Hs.241257	transforming growth factor beta binding protein 1	0.096	0.128	0.124	0.062	11
Hs.287820	Fibronectin 1	1.051	1.157	1.055	0.736	6
Hs.317432	BCAT1	-0.023	-0.012	-0.053	-0.022	144
Hs.5591	MKNK1	-0.082	-0.170	-0.149	-0.149	56
Hs.62	PTPN12	0.111	0.100	0.104	0.126	50
Hs.66581	Protein disulfide isomerase family A, member 2	-0.024	-0.028	-0.034	-0.013	3
Hs.75335	GATM	-0.270	-0.259	-0.250	-0.250	2
Hs.76307	neuroblastoma, suppression of tumorigenicity 1	0.435	0.303	0.616	0.416	4
Hs.78225	NBL1	0.011	0.010	0.018	0.010	64
Hs.83383	Peroxiredoxin 4	-0.074	-0.094	-0.066	-0.085	5

We evaluate the biological implications of selected genes by surveying [29] and other public databases. Among the 15 genes, several have been previously identified in independent studies. Specifically, gene Hs.107 (Fibrinogen-like 1) is a member of the fibrinogen family. In large scale proteomic analysis of serum samples, certain members from the fibrinogen family have been found to be over-expressed in pancreatic cancer samples [30]. Gene Hs.12068 (Carnitine acetyltransferase) is a key enzyme in the metabolic pathway in mitochondria, peroxisomes, and endoplasmic reticulum. CRAT catalyzes the reversible transfer of acyl groups from an acyl-CoA thioester to carnitine and regulates the ratio of acylCoA/CoA in the subcellular compartments. In addition, CRAT has been found to be significantly under-expressed in PDAC samples [31]. Gene Hs.169900 (PABPC4) is localized primarily in the cytoplasm. It may be necessary for the regulation of stability of labile mRNA species in activated T cells. It is one of the pancreatic cancer biomarkers identified in [26], where it is down-regulated at least four-fold in four or more PDAC specimens.

Gene Hs.180920 (RPS9 ribosomal protein S9) encodes a ribosomal protein that is a component of the 40S subunit. The protein belongs to the S4P family of ribosomal proteins. Crnogorac-Jurcevic et al. [32] was the first to identify the association between the dysregulated expression of PRS9 and PDAC. Gene Hs.287820 (fibronectin 1) encodes fibronectin, a glycoprotein present in a soluble dimeric form in plasma and in a dimeric or multimeric form at the cell surface and in the extracellular matrix. Fibronectin plays an important role in maintaining the structural integrity of the pulmonary epithelium and endothelium. Decreases in serum fibronectin and increases in pulmonary leukocyte margination during acute pancreatitis may compromise the integrity of the air-blood barrier and also increase the pulmonary uptake of circulating pathogenic materials. Gene Hs.317432 (BCAT1) encodes the cytosolic form of the enzyme branched-chain amino acid transaminase. This enzyme catalyzes the reversible transamination of branched-chain, alpha-keto acids to branched-chain, L-amino acids essential for cell growth. It is one of the pancreatic cancer markers broadly identified [33]. Gene Hs.5591 (MKNK1) belongs to the MAPK pathway, which has been identified to be associated with the development of multiple cancers. Protein encoded by gene Hs.62 (PTPN12) is a member of the protein tyrosine phosphatase (PTP) family. PTPs are known to be signaling molecules that regulate a variety of cellular processes including cell growth, differentiation, mitotic cycle, and oncogenic transformation. As has been pointed out by [28], gene Hs.75335 (GATM) has been identified as a pancreatic cancer marker in multiple independent studies. Gene Hs.78225 (NBL1) is located at chromosome 1p36. Deletion of material from this region is common in ineuroblastoma. It is possible that a tumor suppressor gene is present in this region.

Ideally, statistical evaluations of the MTGDR should be based on independent data, though it is often unavailable. As an alternative, we conduct evaluations using the following Leave-One-Out (LOO) approach, which has been adopted extensively in cancer microarray studies. We first remove one subject from the dataset. With the reduced dataset, we compute the MTGDR estimate. We note that, to get a relatively fair evaluation, a new set of tuning parameters needs to be computed for the reduced dataset. With the MTGDR, we are able to obtain one regression model for each individual dataset. Then using the model for the dataset that the removed subject belongs to, we are able to predict the probability and class membership (by dichotomizing the predicted probability at 0.5) for the removed subject. We repeat this procedure over all subjects and compute the classification error. With the LOO approach, the MTGDR misclassifies 2 subjects in data P3; otherwise, it achieves perfect classification.

#### Analyses With Alternative Approaches

To facilitate a more comprehensive understanding of the MTGDR approach and the pancreatic study, we conduct the following additional analyses.

#### ANALYSIS WITH THE POOLED TGDR APPROACH

As in the simulation study, we ignore the fact that the four datasets are from different studies that use different platforms. We pool the four datasets and analyze them using the TGDR approach. The sample size of the pooled dataset is 56. A total of 22 genes are identified using this approach. Specifically, this approach identifies 13 of the 15 genes identified by the MTGDR and misses genes BCAT1 and NBL1. As discussed in the above section, both of those two genes have important implications in pancreatic cancer development. (More detailed information on gene identification using this approach is available upon request.) We also evaluate performance of the pooled approach using the LOO. Two subjects (1 in P3 and 1 in P4) are not properly classified.

#### META ANALYSIS BASED ON INDIVIDUAL TGDR

We first analyze each dataset using the TGDR approach and then search for genes identified in multiple studies. This is a voting-based meta analysis approach. For the four datasets, TGDR identifies 7 (P1), 10 (P2), 6 (P3), and 1 (P4) genes, respectively. The numbers of overlaps with genes identified using the MTGDR are 1, 1, 2, and 0, respectively. There is only 1 gene identified with both P2 and P3. Otherwise, there is no overlap between genes identified with the four datasets. Genes identified in one study cannot be used to satisfactorily predict subjects in other studies. For example, we use genes identified in P2 and the corresponding logistic model to make predictions for the rest of the three datasets. Four (P1), 6 (P3), and 4 (P4) subjects cannot be properly classified.

#### META ANALYSIS OF MARGINAL EFFECTS

With the MTGDR and the two alternative approaches, we search for genes with *joint *effects on pancreatic cancer development. To provide a more comprehensive analysis of the pancreatic data, we conduct the following analysis of *marginal *effects. Since the pancreatic data have the "normal versus cancer" binary setup, for each dataset and each gene, we conduct the two-sample comparison of expressions of normal versus cancer samples using the t-test and compute the p-value. For each gene, we combine the p-values across four studies using the Fisher's approach [1]. We then rank genes using the p-values from the meta analysis. Genes with smaller combined p-values have smaller ranks. We note that this is the conventional meta analysis approach for data with binary outcomes. With this approach, we investigate the *marginal *associations between each individual genes and the cancer outcome. We show the ranks for the MTGDR-identified genes in Table 3. We can see that several MTGDR-identified genes have very low ranks. Specifically, genes with marginal ranks 1–7 are identified using the MTGDR. However, there are also MTGDR-identified genes with very high ranks. For example, genes Hs.317432, Hs.5591, and Hs.62 have ranks 144, 56, and 50, respectively. Our analysis suggests that meta analysis and identification of genes with joint effects cannot be replaced with meta analysis of marginal effects.

### Liver cancer study

#### Data

Gene expression profiling studies have been conducted on hepatocellular carcinoma (HCC), which is among the leading causes of cancer deaths in the world. We conduct meta analysis using the four liver cancer microarray datasets described in [2]. Detailed data information is provided in Table 4, where the four datasets are referred to as D1–D4, respectively. The four datasets were generated in three different hospitals in South Korea. Although the studies were conducted in a controlled setting, Choi et al. [2] "failed to directly merge the data even after normalization of each dataset."

**Table 4:** Liver cancer study: Data Information.

Dataset	D1	D2	D3	D4
Experimenter	Hospital A	Hospital B	Hospital C	Hospital C
# tumor	16 (14)	23	29	12 (10)
# normal	16 (14)	23	5	9(7)
Chip type	cDNA(Ver.1)	cDNA(Ver.1)	cDNA(Ver.1)	cDNA(Ver.2)
(Cy5:Cy3)	sample:normal liver	sample:placenta	sample:placenta	sample:sample

Tumor: number of tumor samples. Normal: number of normal samples. Numbers in the "()" are the number of subjects used in the analysis. Ver. 2 chips have different spot locations from Ver. 1 chips.

In studies D1–D3, expressions of 10336 genes were measured. In study D4, expressions of 9984 genes were measured. We focus on the 9984 genes measured in all four studies. For each dataset, the within-print-tip-group normalization is first carried out. We then process the data as follows:

(1) Un-supervised screening:

(1.1) if a gene has more than 30% of missingness in any dataset, it is removed from downstream analysis. In total, 3122 out of 9984 genes pass this screening.

(1.2) if a subject has more than 30% missing expressions for the 3122 genes, then this subject is removed. Eight subjects are removed, leading to an effective sample size of 125. We show the number of subjects actually used in the analysis in Table 4.

(2) For each dataset, we fill in missing expression values with medians across samples.

(3) Supervised screening: for each dataset, we compute the two-sample t-statistic for each gene. We then assign a rank to each gene based on the t-statistic. The overall rank for one gene is defined as the sum of ranks across all four datasets. One thousand genes with the lowest ranks are selected for downstream analysis. This rank-based screening shares similar spirits as the one in [11].

(4) For each dataset, we normalize each gene expression to have zero mean and unit variance.

Gene screening is conducted to exclude genes which are very unlikely to be cancer-associated. Similar procedures have been adopted in [20] and others.

#### MTGDR analysis

We employ the MTGDR approach with optimal tuning parameters selected using the 3-fold cross validation. Thirty-four genes are identified as being associated with the risk of developing liver cancer. We provide information and corresponding estimates for identified genes in Table 5. We draw similar conclusions from Table 5 as from Table 3. We note that, for a very small number of genes, the signs of the four estimates are different. For example, for gene 15.4.E1/Rab9 effector p40, three out of four estimated coefficients are positive, and one is negative. The negative coefficient has a small absolute value and can be caused by random variations. Different signs may suggest conflicting biological conclusions. Without having access to the original experimental setup or a gold standard, we are unable to make further explanations of the conflicting signs. Although those genes have been identified with the MTGDR, they should be interpreted with extreme caution because of those conflicting signs.

**Table 5:** Liver cancer datasets: MTGDR estimates and rank (in meta analysis of marginal effects)

Gene Information	D1	D2	D3	D4	Rank
1.2.F.7/noseq/	-0.076	-0.100	-0.078	-0.035	340
1.3.A.8/clone MGC:5207 IMAGE:2901089	0.147	0.199	0.030	0.054	151
10.1.B.9/cDNA FLJ20844 fis, clone ADKA01904	-0.020	-0.016	-0.002	-0.002	556
11.3.F.6/noseq/	-0.275	-0.519	-0.225	-0.170	259
15.1.G.7/Cyt19 protein (Cyt19), mRNA	0.023	0.019	-0.001	0.009	144
15.2.D.10/EST387826 cDNA	-0.041	-0.031	-0.003	-0.015	17
15.3.E.9/hypothetical protein MGC11287	0.016	0.034	0.015	0.014	131
15.4.E.1/Rab9 effector p40 (RAB9P40), mRNA	0.166	0.243	-0.012	0.083	315
17.2.B.11/ATPase, H+ transporting, lysosomal 9 kD	0.145	0.258	0.108	0.020	110
18.3.F.6/nomatch/	0.072	0.073	0.070	0.045	501
19.1.G.5/Ras association (RalGDS/	0.168	0.176	-0.036	0.042	472
2.2.E.11/triosephosphate isomerase 1 (TPI1), mRNA	0.012	0.012	0.004	0.011	59
2.2.G.10/UDP-glucose pyrophosphorylase 2 (UGP2)	-0.296	-0.274	-0.043	-0.178	126
21.3.A.4/noseq/	0.016	0.011	0.002	0.001	723
23.3.H.1/thioredoxin-like, 32 kD (TXNL)	0.285	0.226	0.066	0.033	252
25.2.A.5/noseq/	0.016	0.014	0.001	0.009	974
26.2.D.2/adipose differentiation-related protein (ADFP)	-0.169	-0.114	-0.219	-0.118	12
26.4.B.5/Human zyxin related protein ZRP-1 mRNA	0.161	0.127	0.042	0.070	118
3.2.E.10/Human G protein-coupled receptor V28 mRNA	-0.707	-0.589	-0.359	-0.375	88
4.1.D.1/multiple endocrine neoplasia I (MEN1), mRNA	-0.086	-0.075	-0.130	-0.090	22
4.2.H.5/solute carrier family 22, member 1	-0.014	-0.120	-0.144	-0.092	38
4.3.C.1/noseq/	-0.058	-0.020	-0.008	0.007	123
4.4.B.9/noseq/	-0.438	-0.670	-0.460	-0.502	3
5.1.A.9/noseq/	-0.001	-0.007	-0.002	-0.001	136
5.1.D.1/malate dehydrogenase 2, NAD (mitochondrial)	0.135	0.043	0.063	0.060	214
6.2.E.3/tubulin, beta polypeptide (TUBB), mRNA/	0.024	0.012	0.004	0.011	33
6.3.B.3/noseq/	0.104	0.104	-0.023	0.015	46
6.4.D.11/non-metastatic cells 2, protein expressed NME2	0.053	0.072	0.020	0.025	61
6.4.F.5/H2A histone family, member Z (H2AFZ), mRNA	0.047	0.062	-0.001	0.042	429
7.3.A.5/nomatch/	-0.329	-0.432	-0.297	-0.222	36
7.3.G.9/guanine nucleotide binding protein, q polypeptide	0.073	0.019	0.049	0.029	153
8.2.B.11/cystatin B (stefin B) (CSTB), mRNA	0.040	0.112	0.051	0.046	884
8.2.D.8/RNA helicase-related protein (RNAHP), mRNA	-0.739	-1.369	-1.002	-1.140	1
8.3.A.7/proline-rich Gla polypeptide 2	-0.001	-0.019	-0.024	-0.026	37

We search public databases for independent evidence of associations between identified genes and liver cancer development. Among the identified genes, gene KIAA0406 is one that constitutes the predictor of PI3 kinase activation. The PI3 kinase signaling pathway is emerging as a promising therapeutic target in a number of cancers as well as inflammation and heart diseases. It has been found in a rat experiment that the mRNA and protein levels of Cyt19 are higher in the liver than in other tissues. Gene Rab9 belongs to the RAS oncogene family, which is activated in multiple cancers. ATPases are a class of enzymes that catalyze the decomposition of adenosine triphosphate (ATP) into adenosine diphosphate (ADP) and a free phosphate ion. This dephosphorylation reaction releases energy, which the enzyme (in most cases) harnesses to drive other chemical reactions that would not otherwise occur. RalGDS is an oncogene and can induce transformation and gene expression by activating Ras, Ral, and Rho mediated pathways. The combination of TPI and an antitumor nucleoside, FTD, not only enhances the antitumor efficacy and decreases the toxicity of FTD, but it also suppresses TP-induced angiogenesis. Protein encoded by ADFP is a major constituent of the globule surface. Increases in mRNA levels are one of the earliest indications of adipocyte differentiation. The Human G protein-coupled receptor has been found expressed in lung, heart, and lymphoid tumor tissues. MEN-1 is a cancer predisposition gene and has been found to be activated in pancreatic, ovarian, and male breast cancers. Polyspecific organic cation transporters in the liver, kidney, intestine, and other organs are critical for the elimination of many endogenous small organic cations as well as a wide array of drugs and environmental toxins. Gene SLC22A1 is one of three similar cation transporter genes located in a cluster on chromosome 6. Mutations of gene TUBB have been found in breast and non-small cell lung cancers. Gene H2AFZ encodes a replication-independent member of the histone H2A family that is distinct from other members of the family. Studies in mice have shown that this particular histone is required for embryonic development and revealed that the lack of functional histone H2A can lead to embryonic lethality. This gene encodes a member of the Asp-Glu-Ala-Asp (DEAD) box protein family. Members of this family are believed to be involved in embryogenesis, spermatogenesis, and cellular growth and division.

We conduct statistical evaluations using the LOO approach described above. The MTGDR misclassifies 6 (D1), 8 (D2), 4 (D3), and 2 (D4) subjects, respectively, which leads to an overall classification error of 0.16. We note that, supervised screening has been conducted prior to analysis. To make a fair evaluation, in the LOO procedure, we carry out the supervised screening for each reduced data (with one subject removed) separately. The possibility of overly optimistic evaluation can be minimized.

#### Analyses with alternative approaches

As for the pancreatic study, we conduct the following analyses using alternative approaches.

#### ANALYSIS WITH THE POOLED TGDR APPROACH

We pool the four datasets, which have a combined sample size of 125, and analyze with the TGDR approach. This pooled approach identifies 24 out of the 34 genes identified by the MTGDR, misses 10, and identifies 10 extra genes not identified by the MTGDR. (Detailed information on gene identification using this approach is available upon request.) We also evaluate the performance of this pooled approach using the LOO. Six (D1), 13 (D2), 11 (D3), and 6 (D4) subjects are not properly classified, which leads to an overall classification error of 0.29.

#### META ANALYSIS BASED ON INDIVIDUAL TGDR

We analyze each individual dataset using the TGDR approach and then search for overlaps of identified genes. For the four datasets, the TGDR identifies 27 (D1), 10 (D2), 20 (D3), and 6 (D4) genes. The numbers of overlaps with genes identified using the MTGDR are 4, 4, 3, and 1. Among the identified genes, one is identified in three datasets, another one is identified in two datasets, and the remainder are identified in only one. Genes identified using one dataset cannot be used to make satisfactory predictions for other datasets. For example, when genes identified with D1 and the corresponding logistic regression model are used to predict subjects in the rest of the three datasets, 20 (D2), 8 (D3), and 6 (D4) subjects cannot be properly classified.

#### META ANALYSIS OF MARGINAL EFFECTS

We conduct meta analysis of the marginal effects as described in the pancreatic cancer study. In Table 5, we show the marginal ranks of the MTGDR-identified genes. A few MTGDR-identified genes also have very strong marginal effects. Specifically, genes with marginal ranks 1 and 3 are identified with the MTGDR. On the other hand, there are several MTGDR-identified genes with very high marginal ranks.

## Conclusion

For many types of cancers, multiple microarray experiments have been independently conducted to search for genes associated with the same clinical outcomes. Early studies have suggested that genes identified from the analysis of a single cancer microarray dataset may have low reproducibility. Among the several possible causes are the small sample sizes and lack of statistical power. A cost effective solution is to pool multiple existing datasets with similar study designs and conduct meta analysis. The merits of meta analysis with cancer microarray data have been established in many early studies and summarized in [6]. In this article, we have developed a new gene selection method in the meta analysis of multiple cancer microarray data.

In terms of methodology, the MTGDR differs significantly from existing approaches. Compared to most existing meta analysis approaches, the MTGDR focuses on the selection of genes with joint effects on cancer and embeds gene selection in estimation. Thus, it can complement existing meta analysis of marginal effects and help to provide a more comprehensive description of the effects of genes. When compared to pooled analysis, the MTGDR allows for experiment-specific regression coefficients. Such a strategy shares similar spirits as the random effects approaches in conventional meta analysis. However, existing random effects approaches are designed for data with a small number of covariates and do not have built-in gene selection mechanisms. The MTGDR advances from such approaches by incorporating gene selection in modeling. It can automatically accommodate different experimental setups, especially different platforms. Compared to intensity approaches that seek for transformations of gene expressions, the MTGDR does not need be conducted on a case-by-case basis. In comparison to classic meta analysis approaches, the MTGDR pools and analyzes raw data instead of summary statistics and can be more informative. In addition, the MTGDR puts more emphasis on gene selection.

Our simulation studies suggest that the MTGDR outperforms the meta analysis approach based on an individual dataset gene selection method. More specifically, it is capable of identifying the same number or more of the true positives with a lower false positive rate. In addition, performance of the MTGDR is relatively insensitive to the increase of the number of genes. Analyses of pancreatic and liver cancer studies suggest that (a) the MTGDR is capable of identifying a small number of genes that show relatively consistent effects on cancer outcomes across multiple studies; (b) many of the identified genes have been confirmed in independent studies. The LOO evaluation generates small classification errors; (c) the gene sets identified by the MTGDR can be considerably different from those identified by alternative approaches. Alternative approaches have inferior performance in terms of inconsistency of identified genes across multiple studies and larger classification errors; and (d) genes identified using the MTGDR may differ significantly from genes with low ranks in the meta analysis of marginal effects.

Despite its significant advancements over existing approaches, our study may have the following limitations. First, in the analysis of the liver data, inconsistent signs for a small number of genes are observed. Such inconsistency is not observed in the pancreatic data analysis or the simulation. It is possible to modify the MTGDR algorithm and force the signs to be the same across multiple studies. For example, for a specific gene, suppose that one gradient is small and negative, and the other three gradients are large and positive. We can add an additional thresholding and set the negative gradient to be zero. We choose to allow inconsistent signs, which may help raise an alarm on the comparability of data and the applicability of the proposed approach when such inconsistency is observed. Second, in our data analysis, we are able to provide partial interpretations of the identified genes. Many of these have been confirmed in independent studies. However, for the liver cancer data, detailed information on several identified genes is not available. Since the focus of this study is to develop a new meta analysis approach, we do not further pursue the biological implications of the analysis results. Third, in the analysis, we evaluate the performance of the MTGDR using the LOO approach. With properly utilized cross validation, the evaluation and comparison with other approaches are expected to be reasonably fair. In standard logistic regression analysis, when the sample size is much larger than the number of genes, there are several other ways of evaluating the fitted model and selected covariates. For example, p-values and *R*^2 ^can be computed. However, we note that the validity of those evaluation criterions is established under the "sample size >> number of covariates" setting and is not applicable to the microarray data, where the number of genes is much larger than the sample size. To our best knowledge, there is still no consensus on evaluation methods with cancer microarray meta analysis.

## Methods

### Data and model

For simplicity of notation, we assume that the same set of *d *genes are measured in all *M *different experiments with *M *> 1. When different sets of genes are measured in different experiments, the MTGDR is still applicable by setting the expressions of missing genes as zero. Note that meta analysis can be less powerful when the number of genes measured in all studies decreases. For 1 ≤ *m *≤ *M*, let *Y*^*m *^denote the clinical outcomes and *Z*^*m *^denote the gene expressions in the *m*th experiment. For each experiment, we assume a regression model *Y*_*m *_~ *ϕ*(*Z*^*m*^' *β*^*m*^), where *β*^*m *^is the regression coefficient, *Z*^*m*^' denotes the transpose of *Z*^*m*^, and *ϕ *is the known link function. By considering the joint modeling of multiple genes, we are able to account for the joint effects of genes on the clinical outcomes.

We assume the same link function *ϕ *across different experiments. This assumption has generally been made in meta analysis. However, we allow for different regression cofficients *β*^*m*^and, hence, different models under different experiments. Such a strategy has been motivated by the fixed effect models in meta analysis [10]. The rationale is that a one unit gene expression change in experiment 1 (say, for example, a cDNA study) may not be equivalent to a one unit change in experiment 2 (say, for example, an Affymetrix study). The regression coefficients, which measure the strength of associations, should be allowed to differ.

We choose data with binary outcomes to describe the proposed MTGDR. We note that this method is also applicable to other types of cancer clinical outcomes, as long as statistical models and objective functions can be properly defined. For experiment *m *and binary outcome, *Y*^*m *^= 1 and *Y*^*m *^= 0 may denote the presence and absence of cancer or two different cancer stages, respectively. We assume the commonly used logistic regression model, which postulates that the logit of the conditional probability *logit*(*P*(*Y*^*m *^= 1|*Z*^*m*^)) = *α*^*m *^+ *Z*^*m*^' *β*^*m*^, where *α*^*m *^is the unknown intercept.

Suppose that there are *n*_*m *_iid observations in experiment *m*. The log-likelihood is:

$$R^{m}(\alpha^{m},\beta^{m}=\sum_{j=1}^{n_{m}}Y_{j}^{m}\log\left(\frac{\exp(\alpha^{m}+\beta^{m^{\prime }}Z_{j}^{m})}{1+\exp(\alpha^{m}+\beta^{m^{\prime }}Z_{j}^{m})}\right)+(1-Y_{j}^{m})\log\left(\frac{1}{1+\exp(\alpha^{m}+\beta^{m^{\prime }}Z_{j}^{m})}\right).$$

Since the intercept *α*^*m *^is usually of little interest, for simplicity, we rewrite *R*^*m*^(*α*^*m*^, *β*^*m*^) as *R*^*m*^(*β*^*m*^).

### MTGDR method

The MTGDR is a gene selection method. It embeds gene selection in the construction of regression models. Gene selection then amounts to identifying the nonzero components of the regression coefficients *β*^*m*^.

Under the present setup, it is natural to make the following assumptions: (S1) The sets of genes with nonzero coefficients (i.e., the identified cancer-associated genes) are the same across different experiments. Under meta analysis, we expect certain comparability of multiple studies. Thus, although data generated under different experiments are not directly comparable, the biological conclusions should be comparable. In other words, we should conclude that the same sets of genes are associated with cancer across different experiments; (S2) Although similar logistic regression models are used to link genes with cancer outcomes in all experiments, the nonzero components of the regression coefficients *β*^*m *^may be not equal across experiments. This assumption is due mainly to the concern of different experimental setups, especially platforms.

#### Algorithm

Let *β *= (*β*^1^, ..., *β*^*M*^) and *R*(*β*) = *R*^1^(*β*^1^) + ... + *R*^*M*^(*β*^*M*^). Here *β *is a *d *× *M *matrix. Let Δ*ν *be a small positive increment, as in ordinary gradient descent searching. In the implementation of this algorithm, we choose Δ*ν *= 10^-3^. Let *β*^*m*^(*ν*) denote the parameter estimate of *β*^*m *^corresponding to *ν*. Let 0 ≤ *τ *≤ 1 be a fixed threshold value. The MTGDR algorithm proceeds as follows.

1. Initialize *β *= 0 (component-wise) and *ν *= 0.

2. With current estimate *β*, compute the *d *× *M *negative gradient matrix *g*(*ν*) = -∂*R*(*β*)/∂*β*, where the (*j*, *m*) element of *g *is $g_{j,m}(\nu )=-\partial R^{m}(\beta^{m})/\partial \beta_{j}^{m}$.

3. Compute the length *d *vector of meta gradient *G*, where the *j*^*th *^component of *G *is $G_{j}(\nu )=\sum_{m=1}^{M}g_{j,m}(\nu )$.

4. Compute the meta threshold vector *F*(*ν*) of length *d*, where the *j*^*th *^component of *F*(*ν*): *F*_*j*_(*ν*) = *I*(|*G*_*j*_(*ν*)| ≥ *τ *× *max*_*l*_|*G*_*l*_(*ν*)|) and *I *is the indicator function.

5. Update the (*j*, *m*) element of *β*: *β*_*j*, *m*_(*ν *+ Δ*ν*) = *β*_*j*, *m*_(*ν*) - Δ*νg*_*j*, *m*_(*ν*)*F*(*ν*) and update *ν *by *ν *+ Δ*ν*.

6. Steps 2–5 are iterated *k *times, where *k *is determined by cross validation.

In Step 1, the MTGDR algorithm starts with the zero estimates (i.e., no gene is identified as cancer-associated). In Step 2, the gradients are computed for each individual dataset. Genes with stronger effects on cancer outcomes will have larger gradients. In Step 3, the meta gradient, which is defined as the sum across different experiments, is computed. It evaluates the overall effects of genes on cancer outcomes across multiple experiments. For example, consider that gene 1 shows only a large positive effect in experiment 1 and no effects in other experiments, whereas gene 2 shows moderate negative effects in all experiments. Then the sum of gradients for gene 2, which measures the overall effect across multiple experiments, may be larger than that for gene 1. Gene 2 is thus more likely to be selected since consistent effects are demonstrated across experiments. In Step 4, a meta threshold vector is computed. With this vector, when a gene is selected, it is selected in all models across multiple experiments. In Step 5, we update the MTGDR estimates for only those selected genes. In addition, by allowing for different gradients across multiple studies, the MTGDR allows for different estimates (and, hence, different models) for different experiments.

The tuning parameters *τ *and *k *jointly determine the property of *β *and the property of gene selection. When *τ *≈ 0, *β *is dense even for small values of *k *(i.e, many genes are selected). When *τ *≈ 1, *β *is sparse for small *k *and remains so for a relatively large number of iterations. But it will become dense eventually. At the extreme, when *τ *= 1, the MTGDR usually updates estimates for a single gene at each iteration, which is similar to the stage-wise approaches. When *τ *is in the middle range, the characteristics of *β *are between those for *τ *= 0 and *τ *= 1. For *τ *≠ 0, gene selection can be achieved with cross-validated, finite *k *by having certain components of *β *exactly equal to zero.

As can be seen, the MTGDR involves only simple calculations and can be programmed with many existing software. In our study, research software has been developed using R and is available at [34].

The MTGDR has been partly motivated by the TGDR [35]. The two approaches share a similar thresholding scheme. However, the MTGDR differs significantly from the TGDR by analyzing multiple datasets. When analyzing a single dataset with the TGDR, the effect of a gene can be represented by a single number – its regression coefficient. However, when multiple datasets are present, the effect of a gene needs to be considered across multiple studies and represented with a vector of regression coefficients. Loosely speaking, the TGDR conducts the selection of individual coefficients, whereas the MTGDR conducts the selection of groups of coefficients. Although intuitively simple, extension from individual selection to group selection has been shown to be highly nontrivial.

#### Tuning parameter selection

We use the V-fold cross validation to select the optimal *k *and *τ*. For *τ *= 0,0.05, ..., 0.95,1, we search over *k *to maximize the V-fold cross validation objective function, which can be defined following [20]. With the V-fold cross validation, partial protection against over-fitting is also provided. In this study, we set *V *= 3, which is due mainly to the small sample size consideration.

#### A graphic demonstration

We use the following numerical example to demonstrate the MTGDR parameter paths. For *m *= 1, 2 and 3, we generate data from $logit(P(Y^{m}=1|Z^{m})=\beta_{1}^{m}Z_{1}^{m}+\beta_{2}^{m}Z_{2}^{m}+\beta_{3}^{m}Z_{3}^{m}+\beta_{4}^{m}Z_{4}^{m}$. In this simulated meta analysis, there are three independent experiments and four genes per experiment. $Z_{i}^{j}$s are generated independently and *N*(0,1) distributed. We set *β*^1 ^= (2.0, 2.0, 0,0), *β*^2 ^= (1.5, 1.5, 0,0) and *β*^3 ^= (1.0, 1.0, 0,0). In all three experiments, only the first two genes are associated with the binary outcomes, and their corresponding coefficients are different. We simulate 50 observations in each experiment.

**Figure 1:**
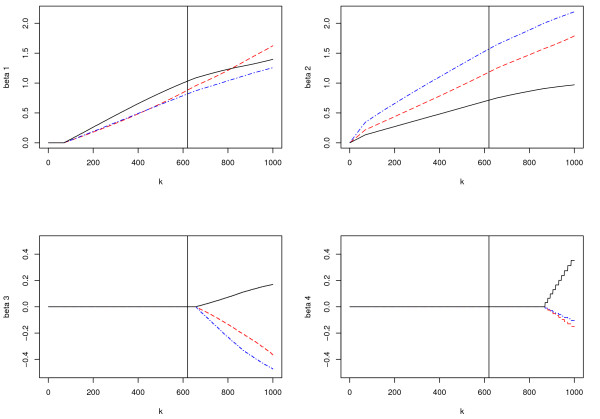
**Parameter paths as a function of *k***. Dashed red line: simulated experiment 1; Dash-dotted blue line: simulated experiment 2; Solid black line: simulated experiment 3. Vertical lines: cross-validated *k*.

The 3-fold cross validation select *τ *= 1.0 and *k *= 620. We show in Figure 1 the parameter paths as a function of *k *for *τ *= 1.0. Individual parameter paths are similar to the stage-wise paths. We can see that for any *k*, the estimated coefficients for one gene are either all zero or all nonzero across experiments. For a specific gene with nonzero coefficients, the estimated coefficients are different across experiments.

## Authors' contributions

Both authors were involved in the study design, data analysis and writing. SM wrote the R code for data analysis. Both authors read and approved the final manuscript.
